# Pretreatment Liver Injury Predicts Poor Prognosis of DLBCL Patients

**DOI:** 10.1155/2017/7960907

**Published:** 2017-09-17

**Authors:** Qing Shi, Rong Shen, Chao-Fu Wang, Xing Fan, Ying Qian, Bin-Shen Ou-Yang, Yan Zhao, Christophe Leboeuf, Anne Janin, Shu Cheng, Li Wang, Wei-Li Zhao

**Affiliations:** ^1^State Key Laboratory of Medical Genomics, Shanghai Institute of Hematology, Shanghai Rui Jin Hospital, Shanghai Jiao Tong University School of Medicine, Shanghai, China; ^2^Department of Pathology, Shanghai Rui Jin Hospital, Shanghai Jiao Tong University School of Medicine, Shanghai, China; ^3^Pôle de Recherches Sino-Français en Science du Vivant et Génomique, Shanghai Rui Jin Hospital, Shanghai Jiao Tong University School of Medicine, Shanghai, China; ^4^U1165 Inserm/Université Paris 7 and Hôpital Saint Louis, Paris, France

## Abstract

Diffuse large B-cell lymphoma (DLBCL) is a heterogeneous group of lymphoma, with different clinical manifestation and prognosis. The International Prognostic Index (IPI), an index designed during the prerituximab era for aggressive lymphoma, showed variable values in the prediction of patient clinical outcomes. The aim of this study was to analyze the prognostic value and causes of pretreatment liver injury in 363 de novo DLBCL patients in our institution. Pretreatment liver impairment, commonly detected in lymphoma patients, showed significant association with poor outcomes and increased serum inflammatory cytokines in DLBCL patients but had no relation to hepatitis B virus replication nor lymphomatous hepatic infiltration. Multivariate analysis revealed that liver dysfunction, advanced Ann Arbor stage, and elevated lactate dehydrogenase (LDH) were independent adverse prognostic factors of both PFS and OS. Accordingly, a new liver-IPI prognostic model was designed by adding liver injury as an important factor in determining IPI score. Based on Kaplan-Meier curves for PFS and OS, the liver-IPI showed better stratification in DLBCL patients than either the IPI or the revised IPI in survival prediction.

## 1. Introduction

Diffuse large B-cell lymphoma (DLBCL) is the most common subtype of non-Hodgkin lymphoma (NHL) [1], while displaying great heterogeneity in clinical manifestation, disease course, and prognosis. The International Prognostic Index (IPI), based on age, performance status, lactate dehydrogenase (LDH), Ann Arbor stage, and extranodal involvements, was originally designed for prediction of prognosis in aggressive lymphoma during the prerituximab era [2]. Although already proven, in a cohort of 2031 patients, it is helpful to stratify DLBCL patients into low-, low-intermediate-, high-intermediate-, and high-risk groups, with 5-year overall survival (OS) rates of 73%, 51%, 43%, and 26%, respectively [2]. Recently, the revised IPI (R-IPI) and National Comprehensive Cancer Network IPI (NCCN-IPI) appear to better predict prognosis in DLBCL patients. The R-IPI identifies three distinct prognostic groups with outcomes categorized as very good (patients with no IPI risk factors, 4-year OS 94%), good (patients with 1 or 2 risk factors, 4-year OS 79%), and poor (patients with 3–5 risk factors, 4-year OS 55%), respectively [3]. The NCCN-IPI is based on five predictors (age, LDH, extranodal sites, Ann Arbor stage, and performance status) and 4 prognostic groups (low (score 0-1), low-intermediate (score 2-3), high-intermediate (score 4-5), and high (score 6–8)). The NCCN-IPI better separates low- and high-risk subgroups (5-year OS: 96% versus 33%, resp.) than the IPI (5-year OS: 90% versus 54%, resp.) [4].

Cytokines are documented to be closely associated with both inflammation and immune modulation while playing a key role in the development of liver damage in a variety of liver disease such as chronic hepatitis B virus (HBV) infection, alcoholic liver injury, nonalcoholic fatty liver disease, and drug-induced liver injury [5–8]. It is generally believed that cytokines are deregulated in many kinds of haematological disorders [9, 10], while elevation of interleukin- (IL-) 6, IL-10, tumor necrosis factor- (TNF-) *α*, IL-8, and IL-2 receptor (IL-2R) was demonstrated valuable in the prediction of unfavorable prognosis in lymphoma [11–14].

The aim of the present study was to determine the role of liver inflammation, reflected by the cytokines and serum transaminase activities, gamma-glutamyltranspeptidase (*γ*-GT), and alkaline phosphatase (ALP) in the prediction of outcome in DLBCL patients.

## 2. Patients and Methods

### 2.1. Patients

We conducted a single-center retrospective case-control study on de novo DLBCL patients. A total of 363 patients were included, with the histological classification confirmed according to the World Health Organization (WHO) 2008 criteria [15]. Serum alanine aminotransferase (ALT), aspartate aminotransferase (AST), *γ*-GT, and ALP were used as markers of liver injury as recommended by the regulatory authorities [16]. Serum cytokine tests (including IL-1*β*, IL-2R, IL-6, IL-8, IL-10, and TNF-*α*) were systematically assessed before chemotherapy. Cytokines were detected in the serum of 15 healthy volunteers as controls. Clinical characteristics of the 363 patients are shown in Table 1. Patients with abnormal liver function, defined as elevation in any of the following four indexes: ALT, AST, *γ*-GT, or ALP, were recruited into the liver dysfunction group; then, a propensity score matching method was used to create the matched control group [17]. Patients were matched at a ratio of 1 : 1 using the nearest neighbor method with a caliber of 0.10. All the patients and volunteers gave their informed consent, following the regulations of the Shanghai Jiao Tong University School of Medicine Institutional Review Boards, in accordance with the Declaration of Helsinki.

### 2.2. Treatment Regimens

340 patients (93.7%) received R-CHOP (rituximab, cyclophosphamide, doxorubicin, vincristine, and prednisone), and 15 patients (4.1%) received CHOP chemotherapy as initial treatment. The rest 8 patients (2.2%) received only palliative care in consideration of the poor performance status or insufficient organ function (Table 1).

### 2.3. Response Criteria

The treatment response was evaluated according to the WHO response criteria [18]. Complete response (CR) was defined as no evidence of residual disease, partial response (PR) as having at least a 50% reduction in tumor burden from the onset of treatment, and no response as having less than a 50% reduction in tumor burden or disease progression. Assessment of the treatment response was evaluated by a follow-up clinical, radiological, or laboratory study, as determined by the clinician, as described previously [19, 20].

### 2.4. Statistical Analysis

Baseline characteristics of patients were analyzed using Student's *t*-tests for continuous variables, *χ*^2^ tests for categorical data, and Mann–Whitney *U* test for the serum level of cytokines. Overall survival (OS) time was measured from the date of diagnosis to the date of death or to the last follow-up. Progression-free survival (PFS) was calculated from the date when the treatment began to the date when the disease progression was recognized or the date of the last follow-up as described previously [19, 20]. Survival functions were estimated using the Kaplan-Meier method and compared by the log-rank test. Univariate hazard estimates were generated with unadjusted Cox proportional hazards. Multivariate survival analysis was performed using a Cox regression model in which significant variables in the univariate analysis were included. *p* < 0.05 was considered statistically significant. All statistical analyses were carried out using Statistical Package for the Social Sciences (SPSS) 22.0 software (SPSS Inc., Chicago, IL, USA).

## 3. Results

### 3.1. Liver Dysfunction in De Novo DLBCL Patients Was Associated with Poor Outcome and High Cytokine Levels in the Serum

Transaminase activities, *γ*-GT, and ALP were measured before chemotherapy in 363 de novo DLBCL patients. Liver injury was observed in 87 patients. The median values of ALT, AST, *γ*-GT, and ALP for those patients with liver dysfunction were 41.0 IU/L (range 10.0 to 577.0), 45.0 IU/L (range 7.0 to 678.0), 65.5 IU/L (range 1.0 to 707.0), and 89.0 IU/L (range 21.0 to 1013.0), respectively, significantly higher than those for the 276 patients without liver dysfunction (*p* < 0.001, Table 1). Patients with liver dysfunction had no relationship with HBV replication or lymphomatous hepatic infiltration but were significantly associated with advanced Ann Arbor stage (*p* < 0.001), poor performance status (*p* < 0.001), increased LDH level (*p* < 0.001), high IPI score (*p* < 0.001), presence of B symptoms (*p* = 0.002), and low CR rate (*p* = 0.004, Table 1). Since cytokines in the serum had been reported to be associated with liver inflammation and dysfunction [5–8], patients with liver dysfunction had significantly higher level of IL-2R, IL-6, IL-10, and TNF-*α* in the serum, when compared with those without liver dysfunction (*p* < 0.001, Table 1).

### 3.2. Liver Dysfunction Was Associated with Poor Outcome and High Serum Cytokine Levels in Matched Case-Control Analysis in DLBCL

To avoid the unfavorable impact of advanced disease stage and high IPI score on the outcome of the patients, 87 of 276 patients without liver dysfunction were selected as case controls using 1 : 1 matching on propensity scores for sex, age, IPI score, and lymphomatous hepatic infiltration, with a caliber of 0.10. Clinical characteristics of the 174 patients selected by propensity score matching are shown in Supplement Table 1S available online at https://doi.org/10.1155/2017/7960907. After matching, elevated LDH level was still observed in patients with liver dysfunction (Table 1S).

**Figure 1:**
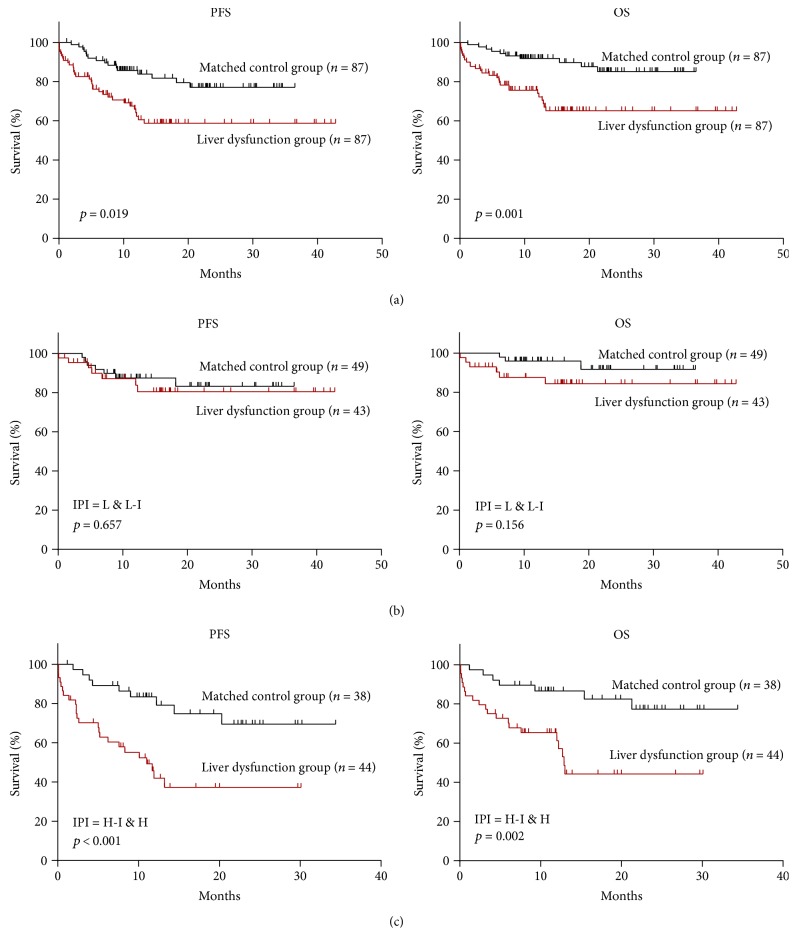
Progression-free survival (PFS) and overall survival (OS) curves based on pretreatment liver function in (a) 174 patients selected by propensity score matching, (b) International Prognostic Index (IPI) low- (L-) and low-intermediate- (L-I-) risk patients, and (c) IPI high-intermediate- (H-I-) and high- (H-) risk patients.

With a median follow-up of 11.7 months in both groups, patients in the liver dysfunction group showed significantly poorer outcomes than those in the matched control group (liver dysfunction group: 2-year PFS 58.5% and 2-year OS 65.2%; matched control group: 2-year PFS 74.0% and 2-year OS 84.9%, *p* = 0.019 and 0.001, resp.; Figure 1(a)).

**Figure 2:**
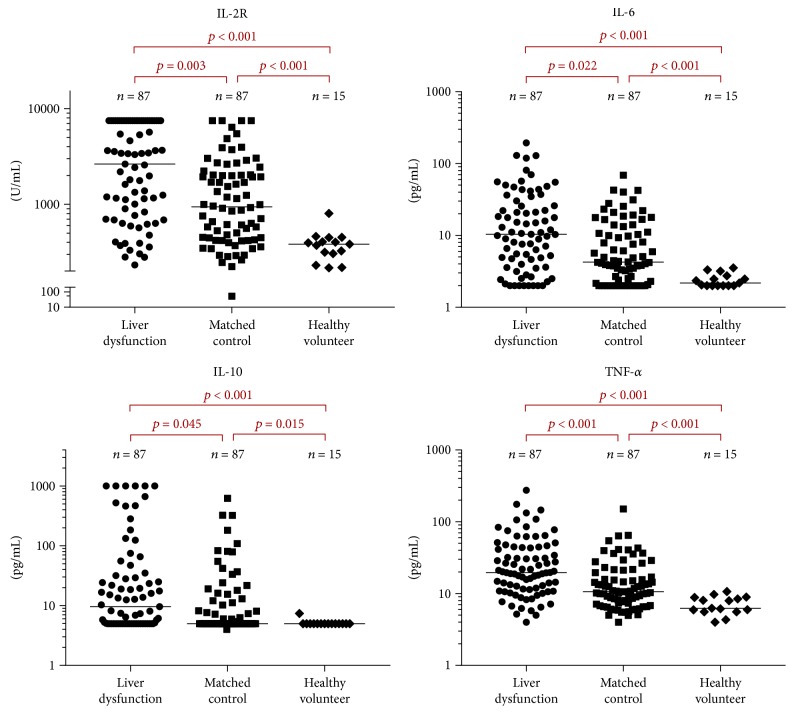
Serum interleukin- (IL-) 2 receptor (IL-2R), IL-6, IL-10, and tumor necrosis factor- (TNF-) *α* levels in the liver dysfunction group, matched control group, and healthy volunteers.

In subgroup analysis according to IPI score, in patients with IPI score 0–2, no significant difference was found for PFS or OS between the matched control group and liver dysfunction group (*p* = 0.657 and *p* = 0.156, resp.; Figure 1(b)). However, in patients with IPI score 3–5, patients in the liver dysfunction group showed significantly shorter PFS and OS when compared with those in the matched control group (*p* < 0.001 and *p* = 0.002, resp.; Figure 1(c)). Of note, patients in the liver dysfunction group retained significantly higher levels of serum cytokines IL-2R, IL-6, IL-10, and TNF-*α*, compared with those in the matched control group (*p* = 0.003, *p* = 0.022, *p* = 0.045, and *p* < 0.001, resp.; Figure 2 and Table 1S) and healthy volunteers (all *p* < 0.001; Figure 2). Interestingly, patients in the matched control group, compared with healthy volunteers, also showed significantly higher serum levels of IL-2R, IL-6, IL-10, and TNF-*α* (*p* < 0.001, *p* < 0.001, *p* = 0.015, and *p* < 0.001, resp.; Figure 2).

### 3.3. Liver Dysfunction Was an Independent Adverse Prognostic Factor by Univariate and Multivariate Analyses in DLBCL

As shown in Table 2, in univariate analysis, decreased OS and PFS rates correlated with high IPI score (both *p* < 0.001), advanced Ann Arbor stage (both *p* < 0.001), poor performance status (both *p* < 0.001), and elevated LDH level (both *p* < 0.001) as well as cytokines IL-2R (both *p* < 0.001), IL-6 (*p* < 0.001 and *p* = 0.004, resp.), IL-10 (both *p* < 0.001), and TNF-*α* (*p* = 0.003 and *p* = 0.005, resp.). Importantly, liver dysfunction was strongly associated with shorter PFS and OS (both *p* < 0.001). Multiple extranodal involvement was of prognostic value only for PFS (*p* = 0.019), and the presence of B symptoms was of prognostic value only for OS (*p* = 0.036).

In multivariate analysis, after incorporating all variables that were significant in univariate analysis, elevated ALT, AST, *γ*-GT, or ALP levels (OR = 1.815, 95% CI 1.075–3.064, *p* = 0.026); advanced Ann Arbor stage (OR = 4.013, 95% CI 2.073–7.769, *p* < 0.001), elevated LDH level (OR = 2.460, 95% CI 1.350–4.482, *p* = 0.003); and IL-6 (OR = 2.460, 95% CI 1.142–5.299, *p* = 0.022) predicted shorter PFS. Similarly, liver dysfunction (OR = 3.352, 95% CI 1.730–6.496, *p* < 0.001), advanced Ann Arbor stage (OR = 3.194, 95% CI 1.435–7.110, *p* = 0.004), and elevated LDH level (OR = 4.404, 95% CI 1.871–10.366, *p* < 0.001) retained their independent prognostic impact on shorter OS (Table 3).

### 3.4. The New Prognostic Model Liver-IPI Was Developed in Our DLBCL Cohort

**Figure 3:**
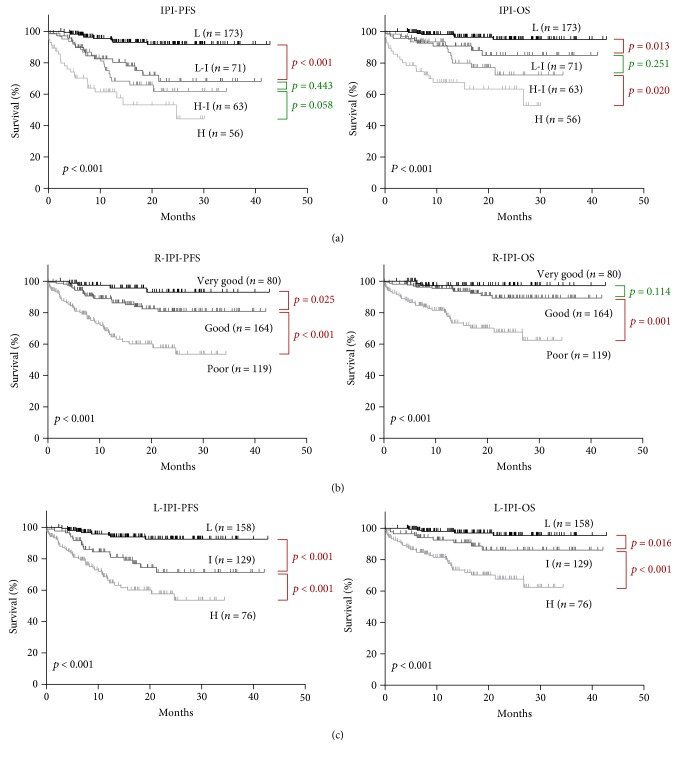
Progression-free survival (PFS) and overall survival (OS) curves according to (a) the International Prognostic Index (IPI), (b) the revised IPI (R-IPI), and (c) the liver-IPI (L-IPI). Four risk groups for IPI score: low- (L-), low-intermediate- (L-I-), high-intermediate- (H-I-), and high- (H-) risk groups. Three risk groups for L-IPI score: low- (L-), intermediate- (I-), and high- (H-) risk groups.

Since liver dysfunction is an independent prognostic factor for both PFS and OS, it was combined with the IPI to design a new prognostic model, named as the liver-IPI. In the liver-IPI model, elevation of ALT, AST, *γ*-GT, or ALP was scored as 1 point, combined with IPI 5 scores to reach a total score of 6. Three risk groups were formed: low-risk (0-1 scores), intermediate-risk (2-3 scores), and high-risk (4–6 scores). The liver-IPI showed better stratification of patients than either the IPI or the R-IPI in OS and PFS, since significant differences were found between low- and intermediate-risk groups (PFS (*p* < 0.001) and OS (*p* = 0.016); Figure 3(c)), as well as in intermediate- versus high-risk groups (*p* < 0.001 for both PFS and OS; Figure 3(c)). However, according to the IPI, no significant difference of OS and PFS was found between the low-intermediate-risk group and high-intermediate-risk group (*p* = 0.251 and *p* = 0.443, resp.; Figure 3(a)). Similarly, no difference of PFS was found between high-intermediate- and high-risk groups (*p* = 0.058; Figure 3(a)). For the R-IPI, there was no statistic difference of OS between the very good and good groups (*p* = 0.114; Figure 3(b)).

## 4. Discussion

To our knowledge, this is the first report showing that pretreatment liver dysfunction was associated with poor prognosis in patients with DLBCL. Elevated serum transaminase activities, *γ*-GT, and ALP were significantly associated with extended lymphoma disease (advanced Ann Arbor stage, elevated LDH level) and alteration of the host status (poor performance status and presence of B symptoms). Meanwhile, it is also revealed that impaired liver function is not directly caused by HBV replication or lymphomatous hepatic infiltration. Of note, in the liver dysfunction group, significant poor treatment outcome with shorter PFS and OS was observed, particularly in those patients of high-intermediate and high risk.

Furthermore, in multivariate Cox regression analysis, pretreatment liver function impairment was an independent unfavorable prognostic factor, which fully demonstrated the prognostic value of liver injury on DLBCL. Therefore, a new prognostic model based on liver function and IPI score, liver-IPI, was designed. The liver-IPI showed a better stratification of different outcomes in patients than the IPI and R-IPI.

In the liver dysfunction group, patients had significantly higher level of IL-2R, IL-6, IL-10, and TNF-*α*, when compared with those in the normal liver function group. Accumulating data has shown that an imbalance in cytokine production is critically involved in the development of liver damage in a variety of liver diseases. TNF-*α*, a central regulator of inflammatory and immune responses, is secreted by activated monocytes, macrophages, and T lymphocytes [21, 22]. Increased TNF-*α* production not only contributes to chronic alcoholic liver injury [23] but also influences the nonalcoholic fatty liver disease process [7]. Soluble IL-2R (sIL-2R) is the soluble form of IL-2R, which is expressed on the cell membrane of lymphocytes and plays an important role in their activation and proliferation [24]. It is released from activated T-cells mainly due to the cleavage by proteinase matrix metalloproteinase-9 produced by inflammation-related cells [25]. The level of sIL-2R reflects the extent of inflammation [26] and correlate with fibrosis stages in patients with chronic HBV infection [5]. Increased IL-6 and IL-10, two major inflammatory cytokines, are reported in ethanol-induced hepatocellular damage and concanavalin A-induced liver injury [27]. *In vivo*, cytokines usually form a network to augment the inflammation and liver impairment. As a mechanism of action, following the induction of IL-6, IL-8, and IL-10 secretion, TNF-*α* could activate the nuclear factor-kappa B pathway and enhance the adhesion molecule expression, which in turn results in adherence of neutrophils and monocytes to the endothelium. Accumulation and activation of inflammatory cells further generate ROS and NO and induce liver damage [8, 28–31]. These mechanisms partially explained the phenomena that pretreatment liver injury was associated with high level of cytokines and poor outcome of patients, without being related to the HBV replication and lymphomatous hepatic infiltration.

Univariate analysis revealed that elevated serum cytokines IL-2R, IL-6, IL-10, and TNF-*α* correlated with the decreased OS and PFS rate. Accumulating researches have pointed out that in lymphoma patients, TNF-*α* accumulation is associated with lymphoma progression [32] and serum sIL-2R is a predictor of poor outcome in DLBCL patients [13, 33]. IL-6 and IL-10 belong to T-helper type 2 cell cytokines, contributing to inhibition of host's immune system and induction of tumor progression [34, 35]. Several studies showed that increased levels of serum IL-6 and IL-10 indicated a poor therapeutic response rate and short survival time in DLBCL [11, 12, 36–38].

## 5. Conclusion

Pretreatment liver injury was an independent poor prognostic factor in newly diagnosed DLBCL patients, correlating with increased serum levels of liver dysfunction-associated cytokines IL-2R, IL-6, IL-10, and TNF-*α*. In addition, liver-IPI, based on liver function and IPI score, had a satisfactory prognostic value in the risk stratification of DLBCL.

## Supplementary Material

Table 1S. Clinical characteristics of matched DLBCL patients (n=174).

## Figures and Tables

**Table 1:** Clinical characteristics of DLBCL patients (*n* = 363).

Characteristics	Liver dysfunction group, *n* (%)	Normal liver function group, *n* (%)	*p* value
Average age (years)	56.7	55.8	0.760
Age (years) > 60	35 (40%)	140 (51%)	0.088
Sex (male)	56 (64%)	156 (57%)	0.195
IPI score			<0.001
Low	26 (30%)	147 (53%)	
Low-intermediate	17 (20%)	54 (19%)	
High-intermediate	20 (23%)	43 (16%)	
High	24 (27%)	32 (12%)	
Ann Arbor stages III-IV	57 (66%)	104 (38%)	<0.001
Number of extranodal sites ≥ 2	35 (40%)	90 (33%)	0.192
Lymphomatous hepatic infiltration	7 (8%)	9 (3%)	0.058
LDH > normal	60 (69%)	94 (34%)	<0.001
Performance status (ECOG) ≥ 2	25 (29%)	27 (10%)	<0.001
Presence of B symptoms	33 (38%)	59 (21%)	0.002
HBV-DNA positive	5 (6%)	8 (3%)	0.213
Hepatitis C virus	1 (1%)	4 (1%)	0.655
Liver enzyme (median values [range], IU/L)			
ALT	41.0 (10.0–577.0)	16.5 (1.0–59.0)	<0.001
AST	45.0 (7.0–678.0)	19.0 (9.0–39.0)	<0.001
*γ*-GT	65.5 (1.0–707.0)	18.0 (1.0–64.0)	<0.001
ALP	89.0 (21.0–1013.0)	69.0 (39.0–122.0)	<0.001
Serum cytokines (median values [range])			
IL-2R (U/mL)	1894.5 (232.0–7500.0)	615.5 (52.1–7500.0)	<0.001
IL-6 (pg/mL)	8.9 (2.0–194.0)	3.6 (2.0–69.1)	<0.001
IL-8 (pg/mL)	43.7 (6.6–3533.0)	54.0 (5.0–2849.0)	0.207
IL-10 (pg/mL)	7.1 (5.0–1000.0)	5.0 (4.0–1000.0)	<0.001
TNF-*α* (pg/mL)	19.2 (4.0–275.0)	9.5 (4.0–151.0)	<0.001
Treatment			<0.001
R-CHOP	75 (86%)	265 (96%)	
CHOP	4 (5%)	11 (4%)	
Supportive care	8 (9%)	0 (0%)	
CR (%)	70.0	85.8	0.004

**Table 2:** Univariate analyses on PFS and OS in DLBCL patients (*n* = 363).

Variates	2-year PFS rate (%)	*p* value for PFS	2-year OS rate (%)	*p* value for OS
IPI score		<0.001		<0.001
Low	91.6		94.9	
Low-intermediate	68.1		85.0	
High-intermediate	61.7		72.1	
High	40.7		50.7	
Ann Arbor stage		<0.001		<0.001
I-II	90.9		93.9	
III-IV	54.3		68.3	
Number of extranodal sites		0.019		0.176
≤1	80.0		85.6	
≥2	62.9		75.6	
Performance status (ECOG)		<0.001		<0.001
≤1	77.8		86.3	
≥2	54.2		60.0	
LDH		<0.001		<0.001
Normal	87.7		94.4	
>Normal	55.0		65.1	
Liver enzyme		<0.001		<0.001
Normal	79.8		88.0	
>Normal	59.5		65.2	
IL-2R		<0.001		<0.001
Normal	90.9		95.1	
>Normal	63.2		74.9	
IL-6		<0.001		0.004
Normal	88.2		91.1	
>Normal	69.2		80.1	
IL-10		<0.001		<0.001
Normal	80.7		87.9	
>Normal	64.0		74.5	
TNF-*α*		0.003		0.005
Normal	88.0		96.5	
>Normal	68.8		76.9	
B symptoms		0.065		0.036
Present	78.0		85.8	
Absent	63.9		72.1	

**Table 3:** Multivariate analyses on PFS and OS in DLBCL patients (*n* = 363).

Variates	PFS	95% CI	*p* value	OS	95% CI	*p* value
Liver dysfunction	1.815	1.075–3.064	0.026	3.352	1.730–6.496	<0.001
Ann Arbor stages III-IV	4.013	2.073–7.769	<0.001	3.194	1.435–7.110	0.004
LDH	2.460	1.350–4.482	0.003	4.404	1.871–10.366	<0.001
IL-6	2.460	1.142–5.299	0.022			
