# Supplementary material for: Pretreatment Liver Injury Predicts Poor Prognosis of DLBCL Patients
Source: Mediators Inflamm. 2017 Sep 17;2017:7960907. doi: 10.1155/2017/7960907 (PMC5646333; doi:10.1155/2017/7960907)
Supplement: Supplementary file 1 — Table 1S. Clinical characteristics of matched DLBCL patients (n=174). [file 7960907.f1.docx]

**Supplement Table**

**Table 1S. Clinical characteristics of matched DLBCL patients (n=174)**

| Characteristics | Liver-dysfunction group,  n (%) | Control matched group,  n (%) | P value |
| --- | --- | --- | --- |
| Average age (years) | 56.7 | 55.2 | 0.832 |
| Age (years) > 60 | 35 (40%) | 41 (47%) | 0.359 |
| Sex (male) | 56 (64%) | 47 (54%) | 0.165 |
| IPI score |  |  | 0.676 |
| Low | 26 (30%) | 31 (36%) |  |
| Low-intermediate | 17 (20%) | 18 (21%) |  |
| High-intermediate | 20 (23%) | 14 (16%) |  |
| High | 24 (27%) | 24 (27%) |  |
| Ann Arbor stage III-IV | 57 (66%) | 48 (55%) | 0.163 |
| Number of extranodal sites ≥ 2 | 35 (40%) | 42(48%) | 0.285 |
| Lymphomatous hepatic infiltration | 7 (8%) | 7 (8%) | 1.000 |
| LDH > normal | 60 (69%) | 42 (48%) | 0.006 |
| Performance status (ECOG) ≥ 2 | 25 (29%) | 21 (24%) | 0.492 |
| Present of B symptoms | 33 (38%) | 25 (29%) | 0.198 |
| HBV-DNA positive | 5 (6%) | 2 (2%) | 0.247 |
| Hepatitis C virus | 1 (1%) | 2 (2%) | 0.500 |
| Liver enzyme (average values [range], IU/L) | | | |
| ALT | 41.0 (10-577) | 16.0 (1-59) | <0.001 |
| AST | 45.0 (7-678) | 20.0 (10-38) | <0.001 |
| γ-GT | 65.5 (1-707) | 19.0 (1-59) | <0.001 |
| ALP | 89.0 (21.0-1013.0) | 68.0 (39.0-122.0) | <0.001 |
| Serum cytokines (median values [range]) | | | |
| IL-2R (U/ml) | 1894.5 (232.0-7500.0) | 1107.5 (52.1-7500.0) | 0.003 |
| IL-6 (pg/ml) | 8.9 (2.0-194.0) | 4.5 (2.0-69.1) | 0.022 |
| IL-10 (pg/ml) | 7.1 (5.0-1000.0) | 5.0 (4.0-1000.0) | 0.045 |
| TNF-α (pg/ml) | 19.2 (4.0-275.0) | 11.3 (4.0-151.0) | <0.001 |
| Treatment |  |  | 0.013 |
| R-CHOP | 75 (86%) | 84 (97%) |  |
| CHOP | 4 (5%) | 3 (3%) |  |
| Supportive care | 8 (9%) | 0 (0%) |  |
| CR (%) | 70.0 | 85.3 | 0.031 |
